# Differential Effects of Olive Leaf Extract on Human Small and Non-Small Cell Lung Cancer and Synergy with Cisplatin: Rationale for Its Potential Use as Food Supplement for Patients?

**DOI:** 10.3390/cells15181666

**Published:** 2026-09-15

**Authors:** Irma Airoldi, Martina Della Lastra, Chiara Brignole, Fabio Morandi

**Affiliations:** 1 UOSD Laboratory of Cell Therapies, IRCCS Istituto Giannina Gaslini, 16147 Genova, Italy; irmaairoldi@gaslini.org (I.A.); martinadellalastra@gaslini.org (M.D.L.); 2 UOSD Laboratory of Experimental Therapies in Oncology, IRCCS Istituto Giannina Gaslini, 16147 Genova, Italy; chiarabrignole@gaslini.org

**Keywords:** lung cancer, olive leaf extract, nutraceuticals

## Abstract

**Highlights:**

**What are the main findings?**

- SCLC cell lines are affected by olive leaf extract (OLE) in a dose-dependent manner in terms of inhibition of cell proliferation and induction of apoptosis, whereas modest effects were observed on NSCLC cell lines;
- OLE activated caspases 3, 7 and 9 only in SCLC cell lines and differentially affected the cellular pathways in SCLC and NSCLC cells.

**What are the implications of the main findings?**

- OLE synergized with cisplatin in the induction of apoptosis in SCLC cell lines;
- OLE is a safe dietary compound, which can be used as a food supplement for patients with lung cancer undergoing chemotherapy.

**Abstract:**

Lung cancer is classified as either Non-Small Cell (NSCLC) and Small Cell Lung Cancer (SCLC) and still represents the leading cause of cancer-related mortality worldwide. Prognosis of NSCLC and SCLC patients is grim; thus, novel therapies are needed. In this context, plant-derived compounds, such as olive leaf extract (OLE), recently attracted much interest for their anti-cancer properties. We investigated the impact of OLE on cell proliferation and apoptosis (by flow cytometry) and migration (by scratch test and transwell assay) of NSCLC and SCLC cell lines, underlying the mechanisms involved. Synergistic effects with cisplatin were also evaluated. OLE differentially impaired cell proliferation and induced apoptosis in LC cell lines. Notably, such effects were higher in SCLC than in NSCLC cell lines. Further analyses were performed on GLC-1 and SKMES cells, identified as OLE-sensitive and OLE-resistant models, respectively. Indeed, OLE triggered the intrinsic apoptotic pathway by activating cleaved caspase-9, -3 and -7, and induced DNA damage response and cellular stress molecules in GLC-1 cells only. In contrast, OLE affected migratory capacity of SKMES cell line and modulated stress adaptation and survival pathways. Finally, OLE synergized with cisplatin in the induction of apoptosis in GLC-1 cells. OLE exerted multiple anti-tumor effects on LC cells in vitro. Its ability to enhance cisplatin-induced apoptosis supports further investigation of OLE as a potential dietary adjunct to chemotherapy. Although OLE has an established safety profile, in vivo studies are required to validate its efficacy, and assess potential interactions with chemotherapeutic drugs before any clinical application.

## 1. Introduction

Lung cancer is the leading cause of cancer-related death globally, accounting for approximately 2.5 million new cases in 2022 [1,2]. Despite progress in systemic therapies the prognosis for patients, particularly in the advanced stages, is poor, with 5-year survival rates of 5–10% [3,4]. Thus, lung cancer remains a global health challenge and novel effective therapies are needed. According to the World Health Organization (WHO) standards [5], lung cancer is broadly divided into two major histological categories based on the appearance of the cells: Non-Small Cell Lung Cancer (NSCLC) and Small Cell Lung Cancer (SCLC) [6,7]. The NSCLC represents the most common form, accounting for approximately 80–85% of all cases [8]. It is further subdivided into three main types that are the adenocarcinoma (the most frequent), the squamous cell carcinoma, and the large cell carcinoma. NSCLC staging uses the TNM system (tumor size, node involvement, metastasis) [5]. According to this system, stage III is particularly complex, as it includes tumors that may be potentially resectable, or unresectable, requiring a multidisciplinary team to decide on the best combination of surgery, radiation, and immunotherapy [9]. The SCLC, accounting for about 10–15% of cases, is a high-grade neuroendocrine tumor characterized by rapid proliferation, early metastasis, and a strong association with a heavy smoking history [10]. Classification is inseparable from staging, which determines how far the cancer has spread. The SCLC staging, predominantly clinical, classifies patients as Limited Stage, when the tumor is confined to one side of the chest, and Extensive Stage, when it has spread beyond the original site. Approximately 70% of SCLC patients are diagnosed at the Extensive Stage [11]. Primary lung cancer in children and adolescence is quite rare, representing only 0.2% of all childhood malignancies. Carcinoid tumor is the most common pediatric form, followed by pulmonary blastoma, mucoepidermoid carcinoma, NSCLC, SCLC, neuroendocrine tumor, squamous cell carcinoma and atypical carcinoma [12,13].

The management of lung cancer is a complex, multidisciplinary effort tailored to the tumor’s histological type and its molecular profile [14]. For decades, platinum-based doublet chemotherapy (e.g., cisplatin or carboplatin combined with agents like pemetrexed, paclitaxel, or gemcitabine) was the standard for advanced disease. While still used, it is now frequently combined with other modalities shifting toward precision medicine and involving targeted therapies harboring actionable genomic alterations (such as EGFR, ALK, BRAF V600E and HER2) and immunotherapy (e.g., immune checkpoint inhibitors with or without chemotherapy) [15,16,17,18,19,20,21]. However, conventional therapy strategy also brings various side effects or toxicity and multidrug resistance problems. Hence, finding alternative natural compounds from plants, potentially increasing the cytotoxic effects of chemotherapy, is of great importance for the treatment of lung cancer.

In this regard, scientific research increasingly views olive leaf extract (OLE) not merely as a simple supplement, but as a sophisticated chemical orchestra where a wide array of phytochemicals works in natural harmony [22]. While oleuropein is the primary bioactive component, representing between 17% and 25% of the extract’s dry weight, the whole plant extract often proves more biologically effective than isolated compounds because its multiple components target various cellular pathways simultaneously [22]. This superiority is largely attributed to the synergy between secoiridoids, simple phenols, and flavonoids, such as luteolin and rutin, which act as supporting instruments that reinforce the pharmacological actions of the main olive phenols [22]. Oleuropein has been shown to effectively inhibit the viability of NSCLC cells (i.e., A549) by inducing G2/M phase cell cycle arrest and triggering the intrinsic mitochondrial apoptotic pathway through the activation of the p38 MAPK signaling cascade [23]. Furthermore, oleuropein was able to induce apoptosis in the same lung cancer cell line A549, by the up-regulation of mitochondrial Glyoxalase 2 (mGlo2), which is driven by superoxide anions and the Akt signaling pathway, while notably sparing healthy, non-malignant cells [24]. Oleuropein also functions as a powerful protective agent against oxidative stress and DNA damage, significantly reducing the migratory potential and invasiveness of alveolar epithelial cells by down-regulating proinflammatory cytokines like interleukin 17A [25]. Nonetheless, no data have been reported regarding the anti-tumor activity of the whole OLE against the A549 cells as well as other types of lung cancers. Of note, evidence documented the ability of OLE to counteract the growth of different human cancer, highlighting the mechanisms involved including: (i) inhibition of cell cycle, (ii) induction of apoptosis, and (iii) inhibition of migration and invasiveness [22]. Beyond direct attack on cancer cells, OLE may also serve as a versatile modulator that can reprogram the immune system to strengthen anti-tumor immunity and counteract the Warburg effect by starving malignant cells of their glycolytic energy source [22,26]. From a clinical perspective, its potential as a chemosensitizer allows it to work in synergy with conventional drugs, such as topotecan in neuroblastoma or cytarabine in pediatric acute lymphoblastic leukemias, potentially enhancing their efficacy while reducing toxic side effects [27,28].

With this background, the aim of our study was to investigate the anti-tumor activities of OLE against human lung tumors in terms of: (i) inhibition of cell proliferation, (ii) induction of apoptosis alone or in combination with cisplatin, (iii) inhibition of cell migration and (iv) modulation of pro- and anti-apoptotic molecules. Our results support the concept that OLE may be a useful nutraceutical instrument for patients affected by lung tumors, thanks to its anti-tumor properties associated with anti-oxidant/immune-stimulating effects and its safety profile.

## 2. Materials and Methods

### 2.1. OLE Composition

OLE was kindly provided by Evergreen Life, San Giovanni al Natisone, Italy. The manufacturer patented a method to extract different active compounds, mainly poliphenols, from olive leaves at very low temperatures. The composition of the product has been previously reported [28], and the concentration of phytochemicals was the following: Oleuropein 2656 mg/L, Hydrohytyrosol 213 mg/L, Tyrosol 174 mg/L, Elenolic Acid 1393 mg/L and Rutin 237 mg/L. Since oleuropein is the most effective and the most concentrated polyphenol in OLE, the concentration of OLE reported throughout the study was referred to the final concentration of oleuropein. We used different batches of OLE throughout the study, with minimal variations in the composition, as disclosed by the manufacturer. The product is stable for a few months at 4 °C.

### 2.2. Lung Cancer Cell Lines

The following human cell lines, 4 NSCLC and 2 SCLC, were used: lung adenocarcinoma cell lines A-549 (ATCC CCL-185), CALU-6 (ATCC HTB-56) and GLC-82 (CVCL_3371), lung squamous cell carcinoma cell line SKMES (ATCC HTB-58), the SCLC cell lines GLC-1 (Cellosaurus CVCL_8200, SCLC) and GLC-4 (Cellosaurus CVCL_0279). Cell lines were purchased from ATCC and Biobank at Ospedale San Martino, Genova, Italy. GLC-1 and GLC-4 cells, growing in suspension, were cultured in RPMI-1640, the other cells grew adherent in DMEM medium (Euroclone, Milan, Italy). Cell lines were kept in culture in an incubator at 37 °C with 5% CO_2_ for no more than 3–4 passages. Media were supplemented with 10% of heat-inactivated fetal bovine serum (FBS, Euroclone), 50 IU/mL penicillin, 50 g/mL streptomycin sulphate, and 2 mM L-glutamine (Euroclone). Fresh medium was replaced every three days.

### 2.3. Cell Proliferation

Proliferation of cell lines was assessed by flow cytometry using CellTrace™ CFSE Cell Proliferation Kit (Thermo Fisher Scientific, Waltham, MA, USA). Briefly, cell lines were resuspended in RPMI-1640 or DMEM without serum and stained with 2 μM Carboxyfluorescein diacetate N-succinimidyl ester (CFSE) at 37 °C for 15 min (min). After washing in RPMI-1640 or DMEM plus 20% FBS, cells were resuspended in medium with 10% FBS and then seeded in 24 well plate (3 × 10^5^/well). Cell lines were cultured with medium alone in the absence (negative controls, CTR−) or presence of FBS (positive control, CTR+), or treated with OLE at the final concentration of 50, 100, 200 and 400 µM. After 6 days of culture, cells were washed and resuspended in 300 μL of MACS buffer (PBS supplemented with 2 μM EDTA and 0.5% BSA) and then analyzed by flow cytometry for cell proliferation, as witnessed by CFSE dilution. Samples were then run on a Gallios^®^ flow cytometer (Beckman Coulter, Brea, CA, USA), acquiring at least 3 × 10^4^ events and data analyzed using Kaluza^®^ analysis software v. 2.0 (Beckman Coulter). Results were expressed as CFSE mean fluorescence intensity (MFI) ± standard error (SE).

### 2.4. Apoptosis

Cells were seeded at 10^5^/well in 96 well plates and cultured for 24, 48 and 72 h (h) with medium alone or in the presence of OLE (50, 100, 200 and 400 µM). Next, apoptosis was analyzed by flow cytometry using the Annexin V-FITC/7AAD kit (Cat#IM3614, lot 300005, Beckman Coulter), following the manufacturer’s instructions. Briefly, 10^5^ cells were washed with PBS and resuspended in 100 μL of binding buffer 1×. Next, 7-AAD and FITC-conjugated Annexin V were added. Cells were incubated for 15 min on ice, then 200 μL of binding buffer 1× were added to each sample and cells were run on a Gallios^®^ flow cytometer (Beckman Coulter), acquiring at least 3 × 10^4^ events. Data were analyzed using Kaluza^®^ analysis software v. 2.0 (Beckman Coulter). Annexin V+ staining identified early apoptotic cells, 7AAD+/Annexin+ the late apoptotic cells and 7AAD+/Annexin- dead cells. Results are expressed as increase in apoptosis calculated as % of cells treated with OLE − % of cells cultured with medium alone. Since this protocol has been validated in previous studies [27,28] and the increase in apoptosis was assessed, a positive control was not included.

### 2.5. Analyses of Cleaved Caspases-3, -7 and -9

The activation of apoptotic pathways was first assessed by evaluating the levels of cleaved caspase-3, -7, -9 in the OLE sensitive GLC-1 and the OLE resistant SKMES cell lines. Cleaved caspase-9 is an initiator caspase involved in the intrinsic mitochondrial apoptotic pathway, whereas cleaved caspase-3 and cleaved caspase-7 are executioner caspases responsible for the proteolytic events leading to apoptotic cell death. Thus, the detection of cleaved caspases by Gallios Flow Cytometer (Beckman Coulter) was used as a marker of apoptotic pathway activation. To this end, cells were cultured in the presence or absence of 200 μM OLE for 48 h and 72 h and commercially available kits from Cell Signaling Technology were used. Cells were washed once with phosphate-buffered saline (PBS) by centrifugation at 300× *g* for 5 min, fixed by adding 100 μL of 4% formaldehyde solution per 1 × 10^6^ cells and incubated for 15 min at room temperature. Cells were then washed with PBS and permeabilized with 90% methanol for 15 min on ice. After washings samples were incubated for 1 h on ice with fluorochrome-conjugated antibodies diluted in Antibody Dilution Buffer according to the manufacturer’s recommendations. The following fluorochrome conjugated rabbit monoclonal antibodies (mAb) from Cell Signaling Technology were used: PE-Cy7 anti-cleaved caspase-3 (Asp175, clone 5A1E, Cat.#9664, lot 2), which detects the large fragment (17/19 kDa) generated following caspase-3 activation; Alexa Fluor 647 anti-cleaved caspase-7 (Asp198, clone D6H1, Cat.#8438, lot 1), which specifically recognizes caspase-7 cleaved at Asp198; and PE anti-cleaved caspase-9 (Asp315, clone D8I9E, Cat.#20750, lot 1), which specifically recognizes caspase-9 cleaved at Asp315.

Isotype-matched mAbs of irrelevant specificity conjugated with the same fluorochrome were used as negative controls. After incubation, cells were washed with PBS and analyzed by flow cytometry, acquiring at least 30,000 events per sample. Data were analyzed using Kaluza software v. 2.0 (Beckman Coulter). The presence or absence of different markers was evaluated as percentage of positive cells and/or mean relative fluorescence intensity (MRFI) calculated as ratio between fluorescence intensity of specific mAb and isotype control.

### 2.6. OLE Molecular Pathways in Lung Cancer Cell Lines

To investigate the molecular pathways involved in the apoptosis induced by OLE in lung cancer cell lines, we selected the GLC-1 cell line for the high response to OLE in terms of inhibition of cell proliferation and induction of apoptosis, and the SKMES cell line for its low response. Cells were treated in the presence or absence of 200 µM OLE for 48 h and protein extracts were obtained following the protocol reported in each human proteome profiler kit. Proteins were quantified by Biorad Protein assay Dye reagents and Tecan Infinite 200 pro Bioreader (absorbance at 555 nm). Four or six hundred µgs of each protein lysate were assessed on 2 different human proteome profiler kits following the manufacturer’s instructions (Biotechne, Minneapolis, MN, USA): (i) apoptosis array (Cat#ARY009, lot P508387) detecting the relative levels of expression of 35 apoptosis-related proteins, and (ii) phospho-kinase array (Cat#ARY003C, lot P481001) with 37 different phosphorylated kinases and 2 related total proteins (i.e., HSP-60 and β-catenin). The relative amount of each individual protein (corresponding to each spot tested in duplicate) was calculated using Image Lab software (Biorad, version 6), upon normalization to internal positive controls. The percentage fold increase was calculated for each protein as follows: [(normalized amount of protein in untreated cells − normalized amount of protein in untreated cells)/normalized amount of protein in untreated cells)] × 100. An arbitrary cut-off value for fold increase/decrease (50%) was established and only proteins with this modulation are shown.

### 2.7. Analysis of Cell Cycle

GLC-1 and SKMES cells were seeded in 6-well plates (2 × 10^5^/well) and the day after treated or not with OLE 200 µM. After 48 h of treatment, cells were pulse-labeled with 10 µM bromodeoxyuridine (BrdU) for 30 min, and then harvested and processed for the evaluation of cell cycle, according to the standard BrdU assay protocol. The detection of BrdU incorporated by the cells during the S-phase of the cell cycle, was carried out by the use of anti-BrdU mAb (MoBU-1, Alexa Fluor™ 488, Invitrogen, Carlsbad, CA, USA). 7-AAD (Beckman Coulter) was used to assess cellular DNA content and allow discrimination of the different cell-cycle phases (G_0_/G_1_, S, and G_2_/M).

### 2.8. Combined Effects of OLE and Cisplatin

Starting from the concept that platinum-based therapy represents the standard for advanced lung cancer, we selected cisplatin to address the question of whether OLE could work in combination with chemotherapy, and produce increased effects against tumor cells. Thus, GLC-1, GLC-4, A549 and SKMES cell lines were treated for 48 h with medium alone, OLE (25, 50, 100 and 200 µM) and cisplatin (Merck, Rahway, NJ, USA 0.625, 1.25, 2.5 and 5 µM) alone or in different combinations, and then analyzed using the Annexin V-FITC/7AAD kit (Beckman Coulter), by flow cytometry. The percentage of apoptotic and alive cells was assessed, as above described. Combined effects of OLE with cisplatin were measured using HSA model for synergistic effects. These analyses were carried out by using SynergyFinder+ web application (https://synergyfinder.org) [29,30,31,32,33].

### 2.9. Migration and Invasion Assays

In order to investigate the potential ability of OLE to affect the migration of lung cancer, we selected the scratch test for the adherent SKMES, A549 and CALU-6 cell lines and the transwell assay for GLC-1 and GLC-4 cells growing in suspension [34].

The latter assay allowed to test the invasive potential of tumor cells in vitro, thanks to cell culture inserts containing an 8 µm pore-size PET membrane with a uniform layer of Matrigel^®^ basement membrane matrix (Corning, Corning, NY, USA). In total, 150.000 GLC-1 or GLC-4 cells in 100 µL of medium (i.e., RPMI 1640 without FBS) were seeded into the upper chamber of 24-well trans-well insert and allowed to settle for 10 min at 37 °C with 5% CO2. Then, cells were treated with 100 µL OLE (final concentration 200 µM) whereas control (CTR) cells did not. Conditioned medium collected from GLC-1 or GLC-4 cultured for 72 h plus 10% FBS was used as chemoattractant and added to the lower chamber (600 µL). After 48 h of incubation, migrated cells in the lower chamber were counted using Turk’s solution.

SKMES, A549 and CALU-6 cells (3 × 10^5^ cells/well) were seeded in 6-well plates (Falcon). Two days after seeding, fresh medium (DMEM 10% FBS with or without 200 µM OLE) was replaced and the cell monolayer was scratched to create a wound, by the use of 200 μL tips. Wound closure was followed over time, by the use of a pticography-based single cell segmentation time-lapse acquisition, by the use of the Livecyte Instrument (Phasefocus, Sheffield, UK). Time lapse lasted for 72 h, with a scan every 1.5 h. Collective migration (μm/hour), area T ½ (hours) and starting area (μm^2^) are shown.

### 2.10. Statistical Analysis

Statistical analysis was carried out using Prism 5.03 software (GraphPad Inc., Boston, MA, USA). Data distribution was analyzed using KS normality test and D’Agostino and Pearson omnibus normality test. Comparison between data sets was analyzed using *t* test or Mann–Whitney test depending on data distribution. *p* values < 0.05 were considered statistically significant.

## 3. Results

### 3.1. OLE Inhibited the Proliferation of Lung Cancer Cell Lines in a Dose-Dependent Manner

**Figure 1:**
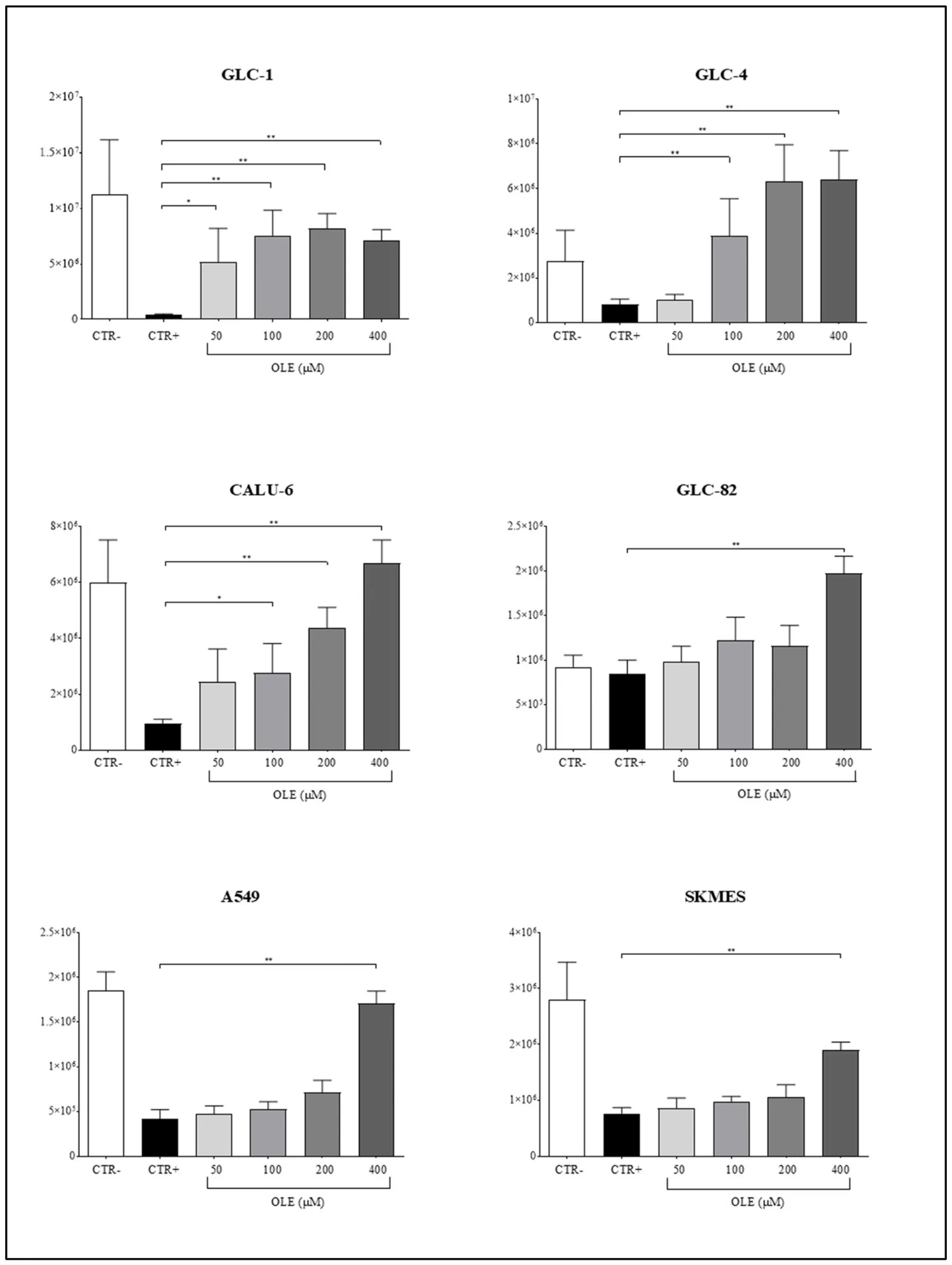
Proliferation of lung cancer cell lines in the presence of OLE. The proliferation of GLC-1, GLC-4, CALU-6, GLC-82, A549 and SKMES cell lines was investigated by CFSE staining and flow cytometry. Cells were cultured for 6 days in medium without FBS (CTR−, white bars), and in complete medium alone (CTR+, black bars) or in the presence of OLE at different concentrations (50, 100, 200 and 400 μM, gray bars). Results are expressed as MFI of CFSE (mean + SE of 5 different experiments) and asterisks indicate statistically significant differences (* *p* < 0.05, ** *p* < 0.005).

We first tested the ability of OLE to modulate the proliferation of six human lung cancer cell lines namely A-549, CALU-6 and GLC-82 (adenocarcinoma), SKMES (squamous cell carcinoma), GLC-1 and GLC-4 (SCLC). Such cell lines were cultured for 6 days in the presence or absence of OLE at increasing doses, ranging from 50 to 400 μM. We observed a different behavior between SCLC and NSCLC since in GLC-1 and GLC-4 there was a statistically significant reduction in proliferating cells at all OLE concentration tested, whereas in NSCLC a clear inhibitory trend was present, with significant growth suppression observed only at the highest concentration (400 µM), with the exception of the CALU-6 cell line that was inhibited starting from 100 µM of OLE (Figure 1). In details, the proliferation of the GLC-1 cell line (upper left panel, CFSE MFI mean ± SE 0.38 × 10^6^ ± 0.1 × 10^6^) was significantly reduced by OLE at 50 μM (5.16 × 10^6^ ± 3 × 10^6^, *p* = 0.02), 100 μM (7.5 × 10^6^ ± 2.2 × 10^6^, *p* = 0.0079), 200 μM (8.16 × 10^6^ ± 1.35 × 10^6^, *p* = 0.004) and 400 μM (7 × 10^6^ ± 1 × 10^6^, *p* = 0.004). Similar data were obtained with the GLC-4 cell line (upper right panel), whose proliferation (0.79 × 10^6^ ± 0.25 × 10^6^) was significantly impaired by OLE at 100 μM (3.88 × 10^6^ ± 1.67 × 10^6^, *p* = 0.04), 200 μM (6.33 × 10^6^ ± 1.62 × 10^6^, *p* = 0.0079) and 400 μM (22.64 ± 2.87, *p* = 0.0079), but not at 50 μM (16.67 ± 3.82). Regarding the NSCLC adenocarcinoma, the cell lines analyzed showed a differential sensitivity to OLE treatment. Indeed, the proliferation of CALU-6 (middle right panel, 0.93 × 10^6^ ± 0.17 × 10^6^) was significantly inhibited in a dose-dependent manner starting from 100 μM (100 μM: 2.77 × 10^6^ ± 1 × 10^6^, *p* = 0.01; 200 μM: 4.35 × 10^6^ ± 0.75 × 10^6^, *p* = 0.004; 400 μM: 6.68 × 10^6^ ± 0.84 × 10^6^, *p* = 0.004). The other adenocarcinoma cells GLC82 (middle left panel) and A549 (lower left panel) were sensitive only to the highest OLE concentration (GLC82: 0.84 × 10^6^ ± 0.15 × 10^6^ vs. 1.97 × 10^6^ ± 0.19 × 10^6^, *p* = 0.004; A549: 0.42 × 10^6^ ± 0.1 × 10^6^ vs. 1.7 × 10^6^ ± 0.13 × 10^6^, *p* = 0.004). SKMES squamous cancer cells (lower right panel, 0.75 × 10^6^ ± 0.1 × 10^6^) showed a very mild profile of responsiveness, with a modest, although significant reduction in cell proliferation only after 400 μM OLE treatment (1.9 × 10^6^ ± 0.13 × 10^6^, *p* = 0.004). Table 1 reports the % inhibition of cell proliferation driven by different concentration of OLE and IC50 values for all cell lines tested.

### 3.2. Differential Effects of OLE on Apoptosis of Lung Cancer Cells

**Figure 2:**
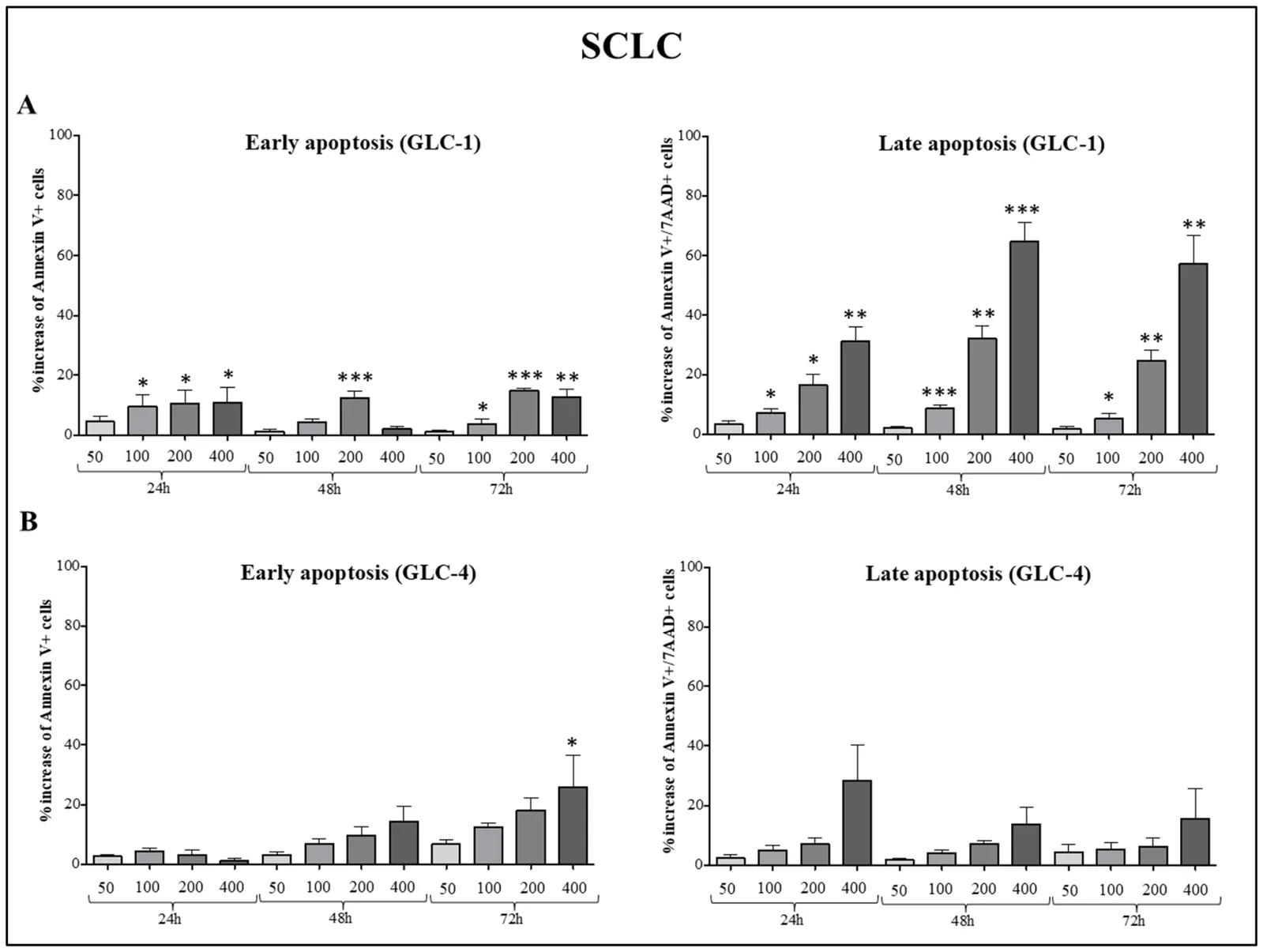
Induction of apoptosis of SCLC cell lines by OLE. The apoptosis of GLC-1 (**A**) and GLC-4 (**B**) SCLC cell lines was investigated by flow cytometry and Annexin V/7AAD staining. Early apoptosis (Annexin V+/7AAD− cells, **upper panels**) and late apoptosis (Annexin V+/7AAD+ cells, **lower panels**) at 24, 48 and 72h are shown. Cells were cultured in complete medium alone or in the presence of OLE at different concentrations (50, 100, 200 and 400 μM). Results are expressed as increase in apoptosis (% of cells cultured in the presence of OLE − % of cells cultured in medium alone, mean + SD of 5 different experiments) and asterisks indicate statistically significant differences (* *p* < 0.05, ** *p* < 0.005, *** *p* < 0.0005).

We next investigated the potential ability of OLE to induce apoptosis in SCLC and NSCLC. To this end, we treated cells with concentration of OLE ranging from 50 to 400 μM at different time points (i.e., 24, 48 and 72 h). These experiments allowed us to highlight that, similar to that reported in the proliferation assays, the GLC-1 SCLC were the most sensitive to OLE treatment compared to all the other cell lines. Indeed, as shown in Figure 2A, OLE induced both early (left panel, AnnexinV+/7AAD−) and late apoptosis (right panel, AnnexinV+/7AAD+) starting from 100 μM at any time point (% increase in early apoptosis, mean ± SE; 24 h:100 μM: 9.4 ± 3.9, *p* = 0.03; 200 μM: 10.5 ± 4.5, *p* = 0.01; 400 μM: 10.9 ± 5, *p* = 0.03; 48 h: 100 μM: 4.2 ± 1.1, *p* = 0.03; 200 μM: 12.2 ± 2.3, *p* < 0.0001; 72 h: 100 μM: 3.7 ± 1.7, *p* = 0.04; 200 μM: 14.7 ± 0.8, *p* < 0.0001; 400 μM: 12.6 ± 2.7, *p* = 0.004; % increase in late apoptosis; 24 h: 100 μM: 7.2 ± 1.3, *p* = 0.03; 200 μM: 16.4 ± 2.5, *p* = 0.02; 400 μM: 30.9 ± 5.1, *p* = 0.006; 48 h: 100 μM: 8.5 ± 1.1, *p* = 0.0006; 200 μM: 32 ± 4.1, *p* = 0.0011; 400 μM: 64.6 ± 6.6, *p* = 0.0002; 72 h: 100 μM: 5.1 ± 1.8, *p* = 0.02; 200 μM: 24.7 ± 3.4, *p* = 0.001; 400 μM: 57.1 ± 9.4, *p* = 0.0019). The other SCLC analyzed (i.e., GLC-4, Figure 2B) appeared to be more resistant and a significant increase in early apoptosis (left panel) was detected exclusively at 400 μM and after 72 h treatment (25.8 ± 10.8, *p* = 0.02). A significant increase in late apoptosis was never observed (Figure 2B, right panel) in these cells.

**Figure 3:**
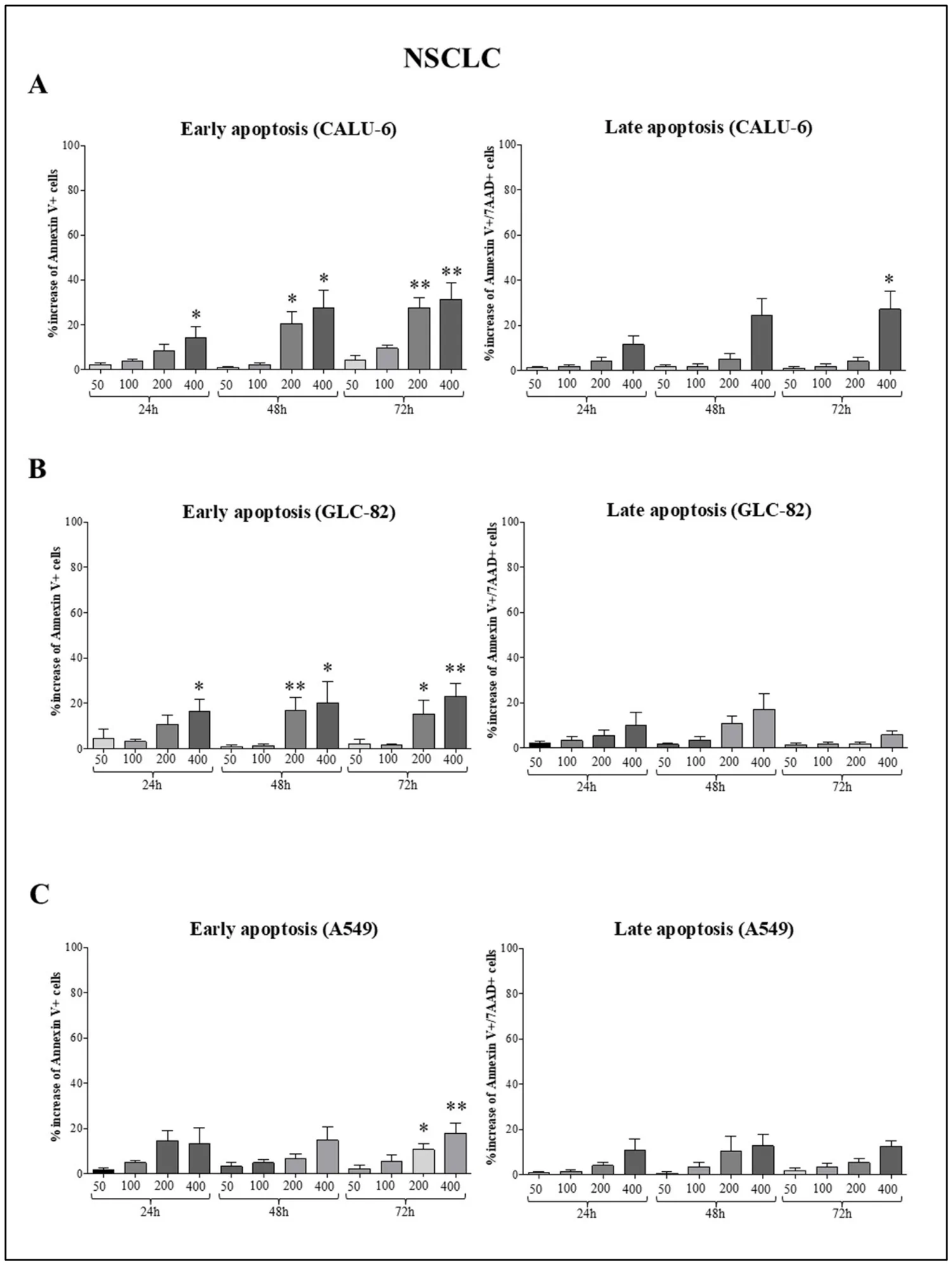
Apoptosis of NSCLC cell lines in the presence of OLE. The apoptosis of CALU-6 (**A**), GLC-82 (**B**) and A549 (**C**) NSCLC cell lines was investigated by flow cytometry and Annexin V/7AAD staining. Early apoptosis (Annexin V+/7AAD− cells, **left panels**) and late apoptosis (Annexin V+/7AAD+ cells, **right panels**) at 24, 48 and 72 h are shown. Cells were cultured in complete medium alone or in the presence of OLE at different concentrations (50, 100, 200 and 400 μM). Results are expressed as increase in apoptosis (% of cells cultured in the presence of OLE − % of cells cultured in medium alone, mean + SD of 5 different experiments) and asterisks indicate statistically significant differences (* *p* < 0.05, ** *p* < 0.005).

The CALU-6 (Figure 3A) and GLC-82 (Figure 3B) NSCLC revealed an induction of early apoptosis (left panels) after 400 μM OLE treatment at 24 h (14.2 ± 5.1, *p* = 0.03 and 16.3 ± 5.5, *p* = 0.04, respectively) and at 200 μM and 400 μM at 48 h (CALU6, 200 μM: 20.2 ± 5.2, *p* = 0.01; 400 μM: 27.7 ± 7.6, *p* = 0.01; GLC82, 200 μM: 16.7 ± 5.9, *p* = 0.009; 400 μM: 20.3 ± 9.4, *p* = 0.03) and 72 h (CALU6, 200 μM: 27.5 ± 4.7, *p* = 0.003; 400 μM: 31.3 ± 7.1, *p* = 0.009; GLC82, 200 μM: 15.2 ± 6.2, *p* = 0.02; 400 μM: 23.1 ± 5.7, *p* = 0.002). However, the late apoptosis (right panels) was significantly induced exclusively in the CALU-6 cells only at the highest OLE concentration after 72 h treatment (27 ± 7.8, *p* = 0.01). The A549 (Figure 3C) cells resulted in poor responsiveness with early apoptosis induced with OLE 200 and 400 μM at 72 h (left panel, 10.6 ± 2.7, *p* = 0.01 and 17.8 ± 4.6, *p* = 0.002, respectively) and without induction of late apoptosis (right panel). Finally, in the SKMES cell no early nor late apoptosis was observed at 24, 48 and 72 h treatment (not shown). Table 2 reports data obtained at 48 h of treatment in all cell lines tested. Taken together, these results demonstrated that the GLC-1 cells are the most respondent to OLE treatment, whereas the SKMES the less responder. For this reason, we used these two lung cancer cell lines for further mechanistic studies.

### 3.3. Effects of OLE on Apoptosis Signaling Networks in GLC-1 SCLC

**Figure 4:**
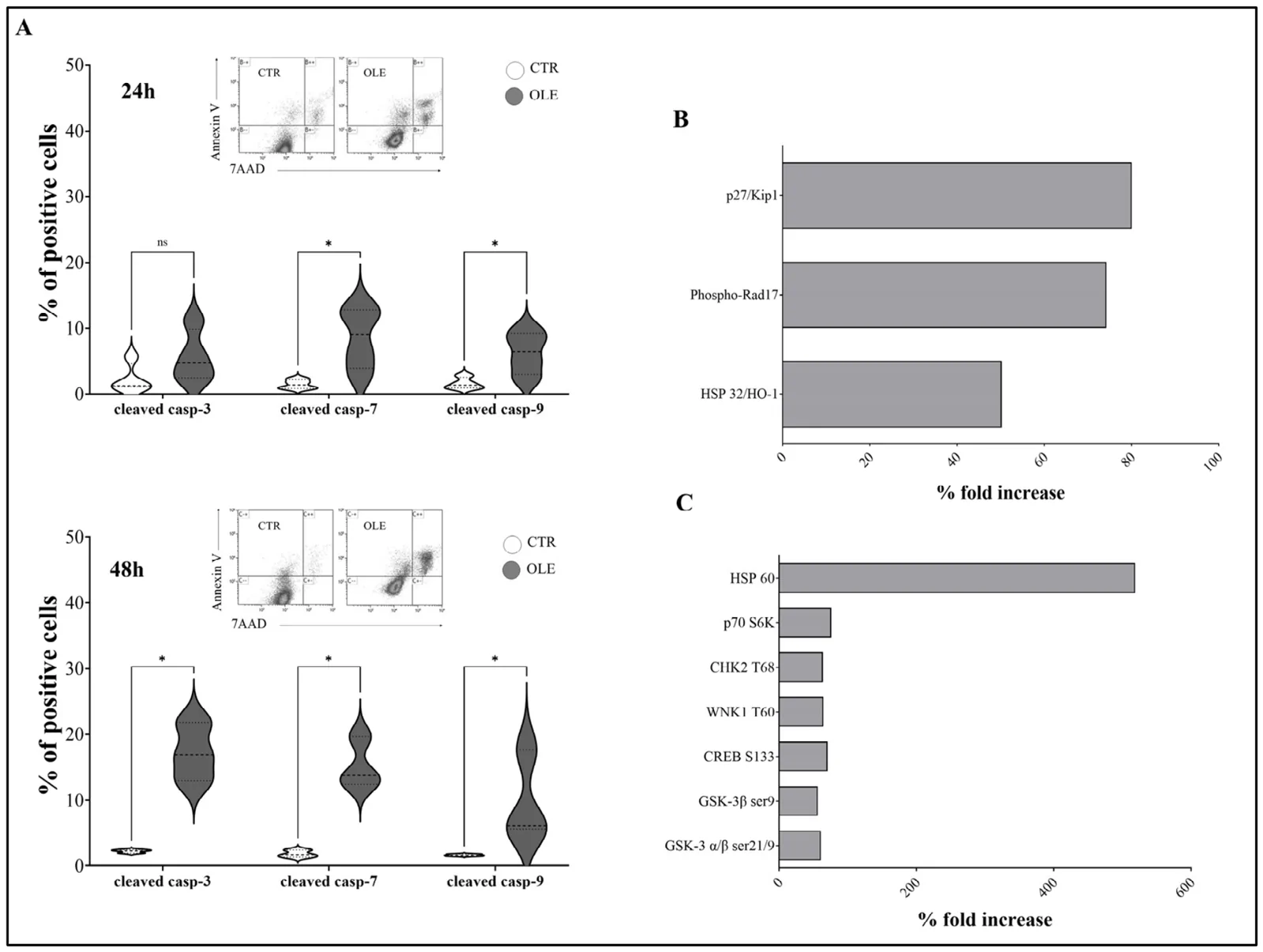
Modulation of apoptotic pathways in GLC-1 cell line by OLE treatment. (**A**). Violin plots show the induction of cleaved caspase -3, -7 and -9 investigated by flow cytometry in GLC-1 cell line cultured in medium alone (white profiles) or in the presence of OLE 200 μM (gray profiles) for 24 (**upper panel**) and 48 h (**lower panel**). Plots indicate maximum, minimum, quartiles and medians (from 4 different experiments), the asterisks indicate statistically significant differences (* *p* < 0.05). Insets show the apoptosis of GLC-1 cells in the same experiment (Annexin V/7AAD staining). Antibody arrays were performed on total protein extracts from GLC-1 cells cultured at the same OLE concentration for 48 h. Data obtained using arrays for apoptosis and phosphorylated kinases are shown in (**B**,**C**), respectively. Data are expressed as percentage fold increase that was calculated for each protein as follows: [(normalized amount of protein in untreated cells − normalized amount of protein in untreated cells)/normalized amount of protein in untreated cells)] × 100. An arbitrary cut-off value for fold increase/decrease (50%) was established and only proteins with this modulation are shown.

In order to deep more insight into the mechanisms underlying the induction of apoptosis driven by OLE, we first investigated the involvement of the caspase cascade. To this end, the GLC-1 cells were treated for 24 and 48 h with OLE 200 μM (i.e., a concentration effectively inducing both early and late apoptosis in these cells) and analyzed, by flow cytometry, for the induction of cleaved caspase-3, -7 and -9. As shown in Figure 4, panel A, OLE was able to trigger the activation of the entire caspase cascade at both timepoints. Notably, OLE significantly up-regulated cleaved caspase-9 (% of positive cells, mean ± SD; 24 h: CTR 1.6 ± 0.9, OLE 6.2 ± 3.2, *p* = 0.01; 48 h: CTR 1.6 ± 0.1, OLE 9.8 ± 6.8, *p* = 0.01), initiating the intrinsic mitochondrial pathway, as well as the executioner cleaved caspase-3 only at 48 h (CTR 2.2 ± 0.2, OLE 17.2 ± 4.4, *p* = 0.0002) and -7 at both timepoints (24 h: CTR 1.5 ± 0.7, OLE 8.6 ± 4.6, *p* = 0.006; 48 h: CTR 1.8 ± 0.5, OLE 15.2 ± 3.8, *p* = 0.0007), confirming that the cell death program was being fully carried out. Results from experiments performed after 24 and 48 h treatment are reported in upper and lower panel, respectively. Insets show the corresponding induction of apoptosis in the same experiments, as assessed by flow cytometry (Annexin V/7AAD staining). Next, we investigated which molecule and cellular pathways may be affected in response to OLE treatment, by performing antibody array analyses on cellular lysates. The two selected arrays included 72 molecules of the apoptosis pathway (Figure 4B) and the phospho-kinases involved in cellular stress (Figure 4C).

Analysis of the apoptotic pathway revealed that OLE induced the expression of p27/Kip 1 (% fold increase, 80.3) known to inhibit cyclin E(A)/CDK2 at G1/S phase [35], phospho-RAD17 (74.2) required for the DNA-damage-induced cell cycle G2 arrest [36], and the cell stress response molecules heat shock protein (HSP)-32 (50.2) and HSP-60 (518.5). Such pro-apoptotic features were accompanied by the phosphorylation of kinases implicated in DNA damage or stress response such as p70 (76.1), C-terminal Src kinase homologue (CHK)2 (64), With-No-Lysine K (WNK)1 (64.7), CAMP Response Element-binding protein (CREB, 70.7) and in cell proliferation and cancer progression. The latter include Glycogen Synthase Kinase (GSK)3α and β phosphorylated in serine 9 (56.5) and 21 (60.9), respectively, and represent an inactivation of the proteins.

### 3.4. OLE Modulation of Signaling Networks in SKMES NSCLC

**Figure 5:**
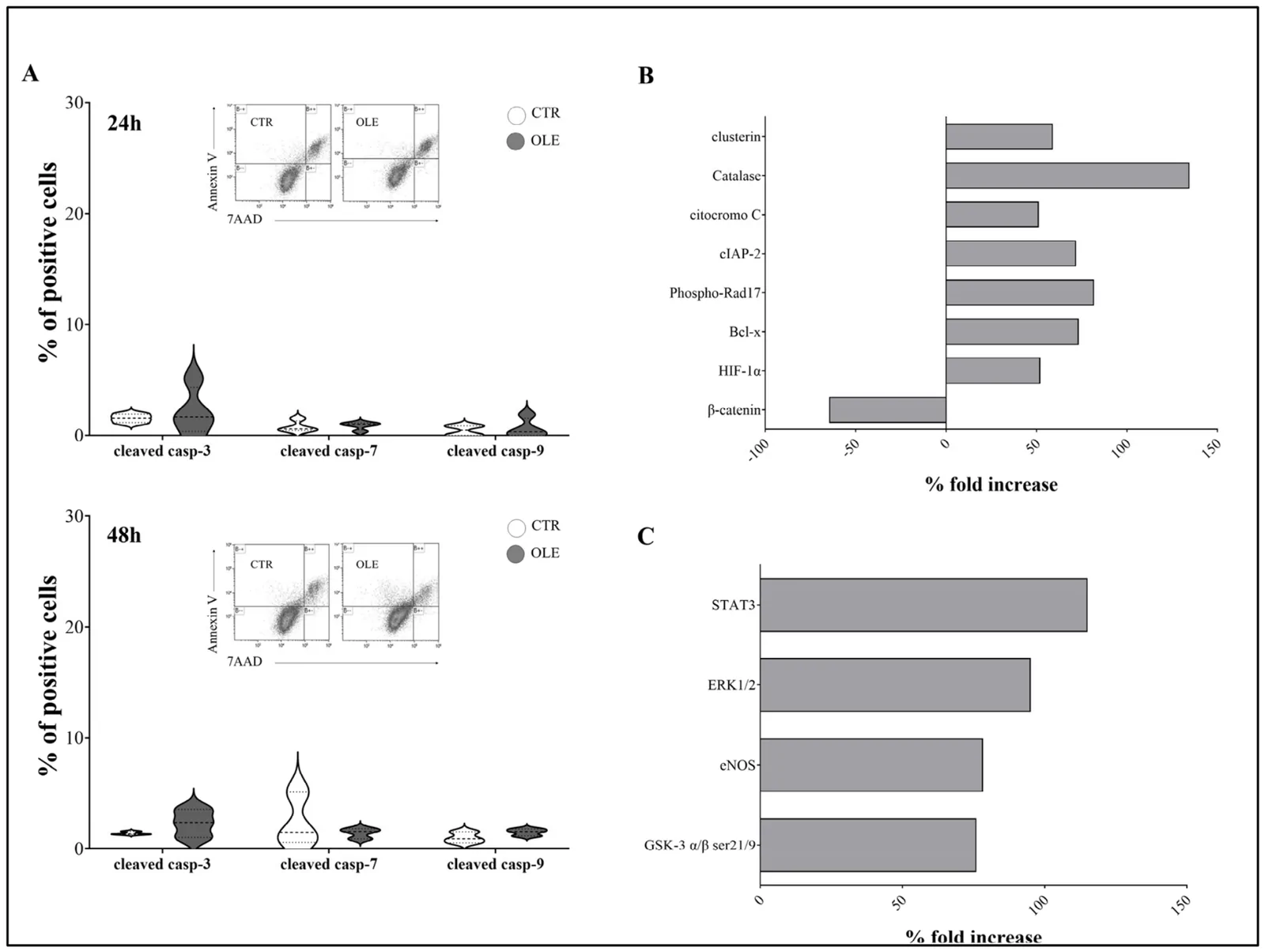
Modulation of the apoptotic pathways in SKMES cell line by OLE. (**A**). Violin plots show the induction of cleaved caspase -3, -7 and -9 investigated by flow cytometry in SKMES cell line cultured in medium alone (white profiles) or in the presence of OLE 200 μM (gray profiles) for 24 (**upper panel**) and 48 h (**lower panel**). Plots indicate maximum, minimum, quartiles and medians (from 4 different experiments). Insets show apoptosis of GLC-1 cells in the same experiment (Annexin V/7AAD staining). Antibody arrays were performed on total protein extracts from SKMES cells cultured at the same OLE concentration for 48 h. Data obtained using arrays for apoptosis and phosphorylated kinases are shown in (**B**,**C**), respectively. Data are expressed as percentage fold increase calculated for each protein as follows: [(normalized amount of protein in untreated cells − normalized amount of protein in untreated cells)/normalized amount of protein in untreated cells)] × 100. An arbitrary cut-off value for fold increase/decrease (50%) was established and only proteins with this modulation are shown.

As mentioned before, the SKMES cells resulted in the most resistant lung cancer cell line to OLE-induced apoptosis, although some effects on cell proliferation were observed. Thus, we tested the signaling networks affected by OLE in terms of cleaved caspase expression by flow cytometry, and modulation of kinase phosphorylation and of apoptotic pathway by antibody array. As reported in Figure 5A, in accordance with the results obtained by flow cytometry using the Annexin V-FITC/7AAD kit, cleaved caspase-3, -7 and -9 were never found to be significantly induced by 24 or 48 h OLE treatment. Accordingly, no induction of apoptosis was observed in the same experiments (insets). Nonetheless, different apoptotic molecules (Figure 5B) and phospho-kinases (Figure 5C) appeared to be modulated. In particular, consistent with the OLE anti-oxidant properties, we found the up-regulation of clusterin (% fold increase, 59) and catalase (134.5), that was parallelled by the induction of molecules involved in cell proliferation and apoptosis including citocrome C (51.2), cellular inhibitor of apoptosis (cIAP, 71.9), phospho-RAD17 (81.7), and Bcl-x (73.3). In addition, hypoxia-inducible factor (HIF)-1α, a molecule with pro-angiogenic activity, was increased upon OLE treatment (52.1) whereas the β-catenin adhesive junction protein decreased (−64.7).

Regarding the kinase pathway, the SKMES cells responded to OLE treatment by phosphorylating, thus activating, Signal Transducer and Activator of Transcription (STAT)3 (115), Extracellular signal-Regulated Kinases ERK1/2 (95), endothelial Nitrox Oxigen (NO) synthase (eNOS, 78.3) with pro-survival effects, but also GSK3α/β (75.9), be-coming inactivated. Different from that observed in GLC-1 cells, WNK1 was found to be reduced upon OLE treatment (−50.3).

### 3.5. OLE Differentially Affects Cell Cycle in GLC-1 and SKMES Cells

**Figure 6:**
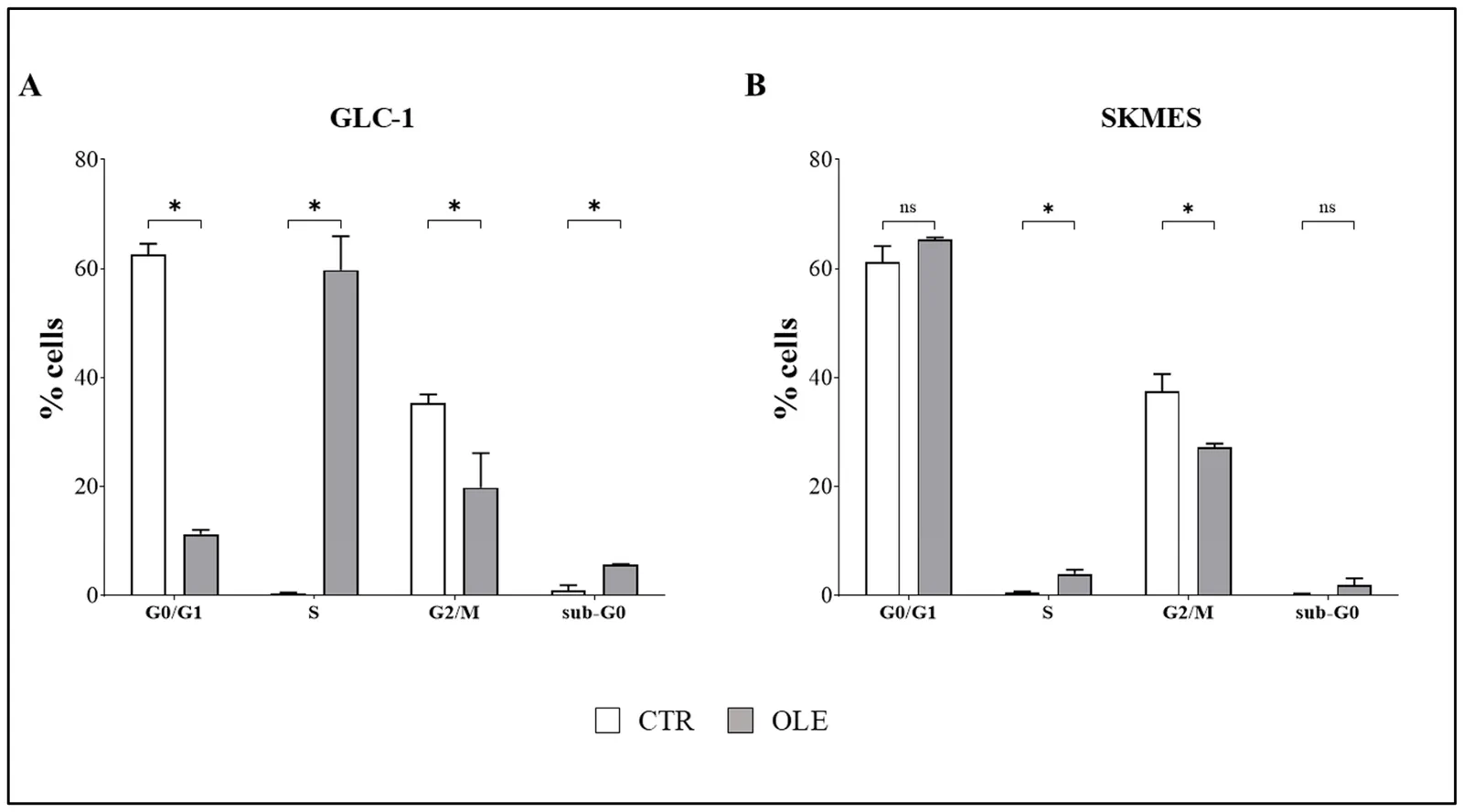
Modulation of GLC-1 and SKMES cell cycle by OLE. Cell cycle was analyzed by flow cytometry using BrdU and 7AAD staining in GLC-1 (**A**) and SKMES (**B**) cell lines cultured in medium alone (white profiles) or in the presence of OLE 200 μM (gray profiles) for 48 h. Data are expressed as percentage of cells in each cell cycle phase (G0/G1, S, G2/M and sub-G0). Mean of 3 different experiments ± SD is shown. The asterisks indicate statistically significant differences, ns means not significant.

We next evaluated the cell cycle of GLC-1 and SKMES cells treated or not with OLE 200 μM. As shown in Figure 6, panel A, GLC-1 cells treated with OLE showed a marked and significant reduction in the percentage of cells in G0/G1 (% of cells ± SD; CTR: 62.06 ± 1.94, OLE: 11.26 ± 0.77, *p* < 0.05) and G2/M (% of cells ± SD; CTR: 35.2 ± 1.66, OLE: 19.7 ± 6.33, *p* < 0.05) phase, paralleled by a strong and significant increase in cells in S (% of cells ± SD; CTR: 0.35 ± 0.16, OLE: 59.7 ± 6.2, *p* < 0.05) and, to a lesser extent, in sub-G0 (% of cells ± SD; CTR: 0.96 ± 0.9, OLE: 5.6 ± 0.13, *p* < 0.05) phase. In contrast, as shown in Figure 6 panel B, the SKMES cells treated with OLE showed only a modest reduction in the percentage of cells in G2/M phase (% of cells ± SD; CTR: 37.4 ± 3.17, OLE: 27.1 ± 0.72, *p* < 0.05) and a very limited increase in cells in S phase (% of cells ± SD; CTR: 0.51 ± 0.2, OLE: 3.97 ± 0.76, *p* < 0.05). The percentage of cells in G0/G1 (% of cells ± SD; CTR: 61.1 ± 2.95, OLE: 65.3 ± 0.39) and sub-G0 (% of cells ± SD; CTR: 0.19 ± 0.15, OLE: 1.92 ± 1.21) phase was not significantly altered by OLE treatment.

These data demonstrated that OLE induced a block of cell cycle through the reduction in cells in G0/G1 phase paralleled by an increase in cells in S phase. In SKMES cells, although a similar behavior to that found on GLC-1 was observed, the extent of the cell cycle arrest was limited, supporting their resistance to OLE treatment, in terms of both cell proliferation inhibition and induction of apoptosis.

### 3.6. Synergistic Pro-Apoptotic Effect of OLE and Cisplatin

**Figure 7:**
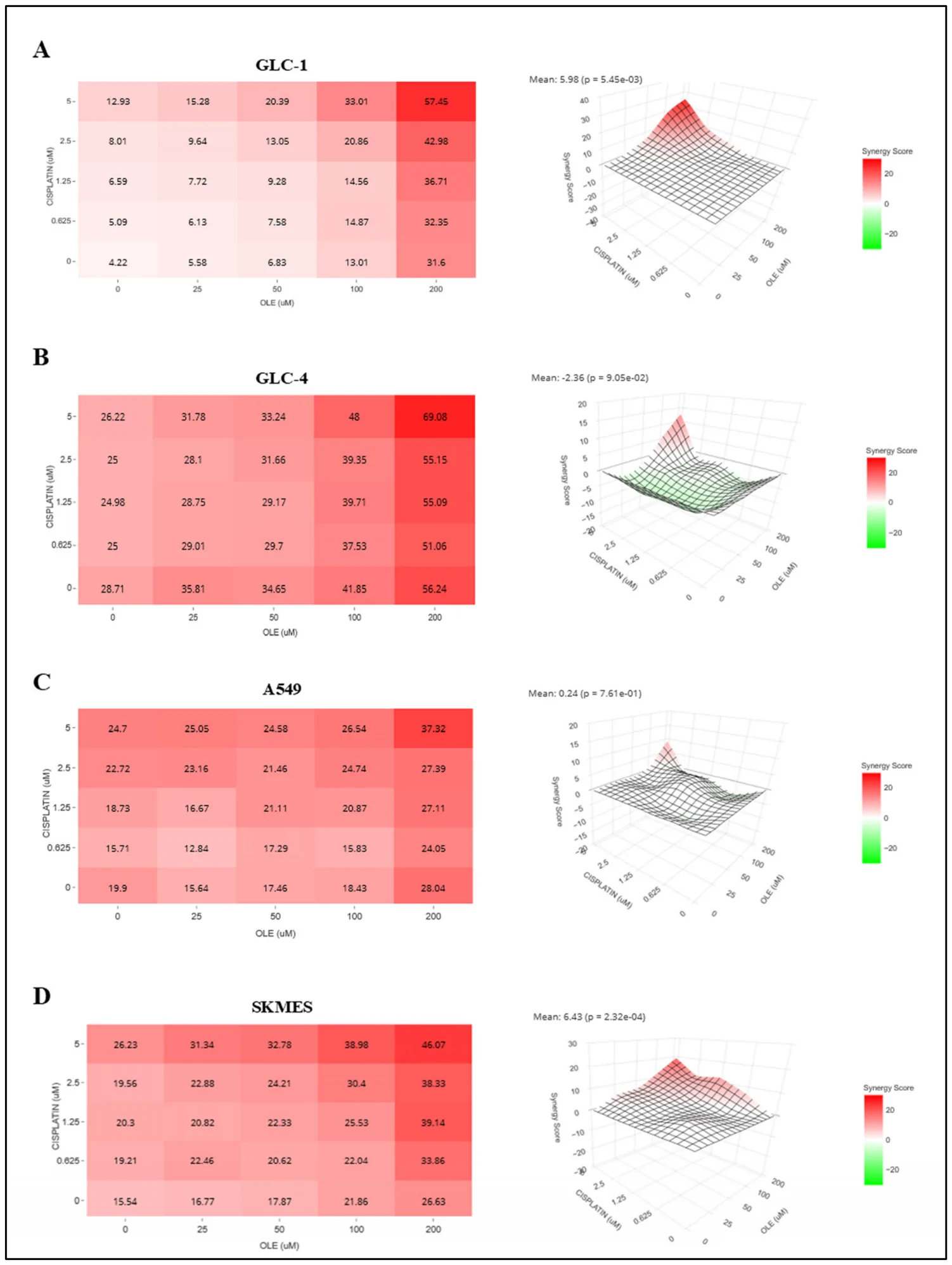
Combinatory effects of OLE and cisplatin. The apoptosis of GLC-1 (**A**), GLC-4 (**B**), A549 (**C**) and SKMES (**D**) cell lines cultured for 48 h with (i) medium alone, (ii) OLE (25, 50, 100 and 200 μM), (iii) cisplatin (0.625, 1.25, 2.5 and 5 μM) and (iv) different combinations of OLE and cisplatin was investigated by flow cytometry and Annexin V/7AAD staining (6 different experiments). Total apoptosis (Annexin V+/7AAD−, Annexin V+/7AAD+ and Annexin V−/7AAD+ cells) was calculated in each experimental condition. Data are reported in the heatmaps in the (**left panels**). Synergistic effects were analyzed by HSA model (**right panels**) where scores and *p* values are reported.

We evaluated the potential additive and/or synergistic pro-apoptotic activity of OLE in combination with chemotherapeutic drugs commonly used to treat lung tumors. To this end, we selected cisplatin as standard-of-care chemotherapeutic agent that was used at the concentrations of 0.625, 1.25, 2.5 and 5 µM. At the same time, GLC-1, GLC-4, A549 and SKMES cells were treated alone or in combination with OLE at 50, 100 and 200 μM. The percentage of apoptotic cells was evaluated by flow cytometry for each experimental condition, and data were analyzed using the SynergyFinder+ web application. The heatmap in left panels of Figure 7 reports the percentage of apoptotic cells evaluated for each experimental combination with means. The synergistic and additive effects were analyzed by the HSA model (right panels). A score > 0 indicated synergistic effect, whereas 0 indicated addictive effect and a score < 0 indicated antagonism. HSA model highlighted a significant synergistic effect of OLE in combination with cisplatin for GLC-1 cell line (score 5.98, *p* = 0.00545) and surprisingly for SKMES cell line (score 6.43, *p* = 0.000232), whereas no synergistic effect was observed for GLC-4 and A549 cell lines.

### 3.7. OLE Impaired the Migration of NSCLC Cells

**Figure 8:**
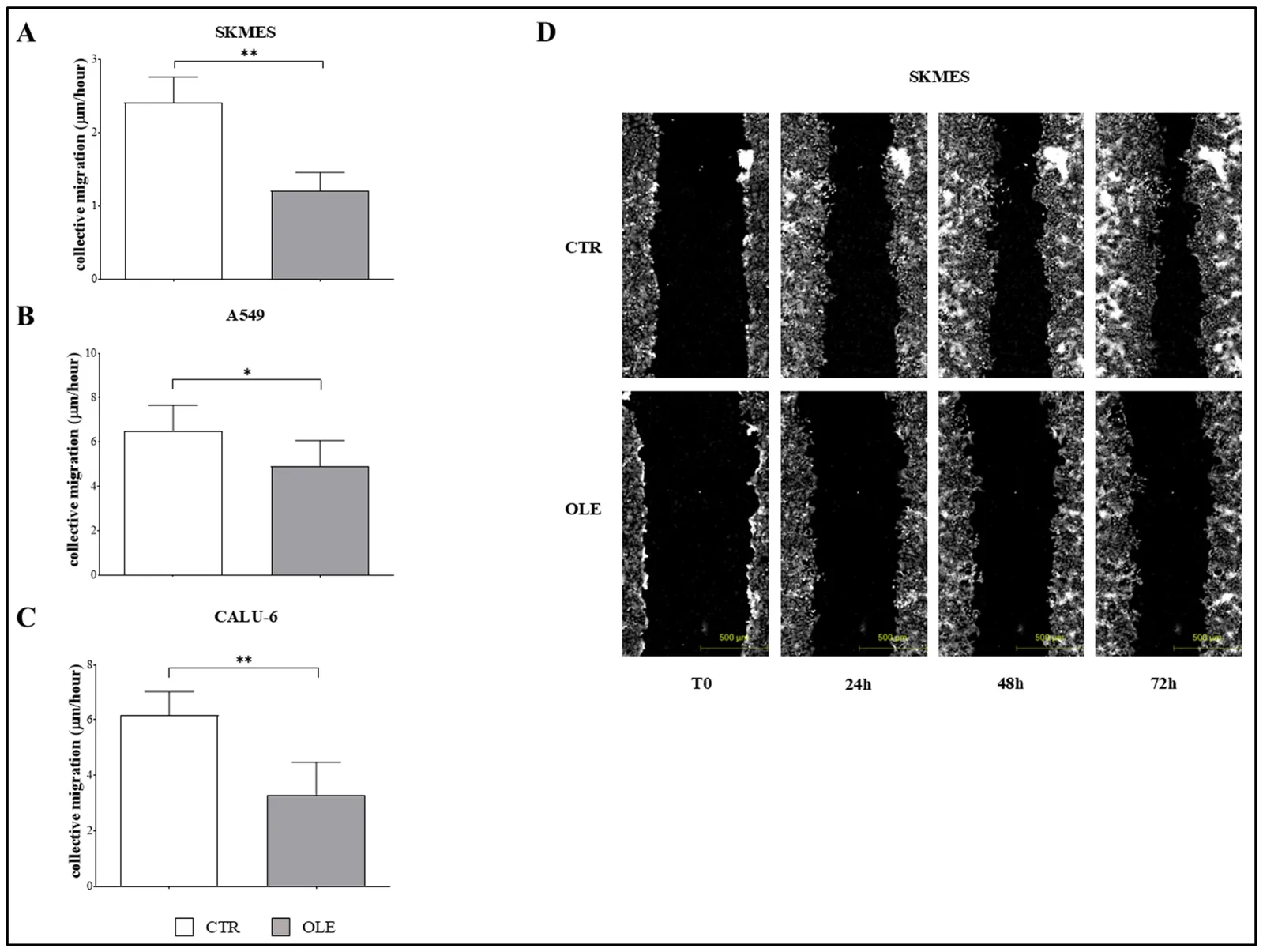
Effects of OLE on the migration of NSCLC cells. Histograms show the collective migration (expressed as μm/hour) of SKMES (**A**), A549 (**B**) and CALU-6 (**C**) cells cultured in medium alone (white bars) or in the presence of OLE 200 μm (gray bars). Results are expressed as mean ± SD of 4 different experiments, asterisks indicate statistically significant differences (* *p* < 0.05, ** *p* < 0.01). (**D**) shows representative images taken at different timepoints (T0, 24 h, 48 h, 72 h) of SKMES cells cultured with medium alone (CTR, **upper panels**) or with OLE 200 μm (OLE, **lower panels**).

The effects of OLE on the migration of NSCLC cells was tested using a scratch test, as previously reported [28,34]. The migration of the cells was followed in real-time, by the use of the Livecyte Instrument, which allows for single-cell segmentation analysis. As shown in Figure 8, OLE (200 uM) was able to inhibit the migration of NSCLC cells, as underlined by the significant reduction in the collective migration (mean rate of closure of the scratch, μm/hour, mean ± SD) of SKMES (panel A, CTR: 2.4 ± 0.35 vs. OLE:1.2 ± 0.27, *p* = 0.005), A549 (panel B, CTR: 6.5 ± 1.1 vs. OLE: 4.9 ± 1.1, *p* = 0.03) and CALU-6 (panel C, CTR: 6.1 ± 0.88 vs. OLE: 3.3 ± 1.1, *p* = 0.001). Figure 8D shows representative images, taken at different time points (T0, 24 h, 48 h, 72 h), of SKMES cells cultured in medium alone (upper panels) or in the presence of OLE (lower panels). In contrast, the migration and invasiveness of GLC-1 and GLC-4 cell lines was nearly undetectable, as assessed by the trans-well assay, but superimposable results were obtained in the presence or absence of OLE (data not shown).

## 4. Discussion

The present study demonstrates that olive leaf extract (OLE) exerts significant anti-tumor activity against human lung cancer cells through the modulation of multiple signaling pathways involved in: (i) cell proliferation, (ii) apoptosis, (iii) migration, and (iv) stress adaptation. Importantly, our results reveal marked differences in OLE responsiveness among lung cancer subtypes, with SCLC cells displaying greater sensitivity than NSCLC cells. Notably, a previous study demonstrated that OLE did not alter the cell viability of normal epithelial cells from small airways, and exerted a protective effect on such cells during inflammation by reducing the production of proinflammatory cytokines [37]. Among the models investigated, GLC-1 cells emerged as the most responsive to OLE treatment, whereas SKMES cells exhibited a pronounced resistance to OLE treatment, resulting in mild inhibition of cell proliferation as well as apoptosis induction. Nevertheless, also in this poor-responsive cell line, alterations in several intracellular pathways were observed. These two cell lines were used for functional studies, as representative models of SCLC and NSCLC, respectively. Indeed, GLC-1 carried typical genetic alterations of SCLC, such as chromosome 3p deletion (which leads to the loss of onco-suppressor genes), and expressed typical markers of SCLC, such as neuron-specific enolase (NSA) and BB isoenzyme of creatine kinase (CK-BB) [38]. On the other hand, SKMES carries a p-53 mutation typical of NCSLC, along with a peculiar expression of different cytokeratins [39,40]. One of the major findings of this study is the potent anti-proliferative and pro-apoptotic activity of OLE in the SCLC GLC-1 cells. The induction of apoptosis was associated with activation of cleaved caspase-9 which starts the intrinsic (mitochondrial) pathway through the activation of the executioner caspases -3 and -7 (which are common to the two distinct apoptotic pathways). These observations are consistent with previous reports describing the ability of olive-derived polyphenols, particularly oleuropein, to induce mitochondrial dysfunction and caspase-dependent apoptosis in different tumor models [22,23,24]. However, our study extends these findings by demonstrating that the whole OLE, containing a complex mixture of bioactive phytochemicals, exerts profound anti-tumor effects in lung cancer cells, supporting the hypothesis that synergistic interactions among multiple olive phenols may enhance their biological activity. Mechanistically, the molecular profile observed in GLC-1 cells suggests that OLE triggers a coordinated cellular response involving cell-cycle arrest, DNA damage signaling, and stress-induced apoptosis. Among the most relevant molecules modulated by OLE, p27/Kip1 was markedly increased. As a critical cyclin-dependent kinase inhibitor, p27/Kip1 negatively regulates G1/S cell-cycle progression and is frequently down-regulated in aggressive tumors [35]. Its induction is therefore consistent with the strong anti-proliferative activity observed in GLC-1 cells and suggests that OLE interferes with cell-cycle progression before the activation of cell death programs. This hypothesis was confirmed by cell-cycle analysis, which demonstrated a block of such cells treated with OLE in S phase. In parallel, OLE induced the phosphorylation of RAD17 and CHK2, two central components of the DNA damage response machinery. RAD17 plays a key role in checkpoint activation following genomic stress [36], whereas CHK2 acts as a downstream effector coordinating cell-cycle arrest, DNA repair, and apoptosis [41]. The concomitant activation of these molecules strongly suggests that OLE is perceived by GLC-1 cells as a source of cellular stress capable of activating checkpoint signaling pathways. Additional evidence supporting the induction of cellular stress derives from the increased expression of HSP-32/HO-1 and HSP-60 [42,43,44]. Although these proteins are often regarded as cytoprotective mediators, their induction may also reflect a cellular attempt to counterbalance oxidative and proteotoxic stress. In the context of GLC-1 cells, the activation of stress–response proteins occurred simultaneously with checkpoint activation and caspase cleavage, indicating that adaptive mechanisms were insufficient to prevent apoptosis. This interpretation is further supported by the modulation of additional stress-responsive kinases, including WNK1 and CREB, which have been implicated in the regulation of cell survival, metabolic adaptation, and responses to environmental stress [45,46]. Interestingly, OLE also induced phosphorylation of GSK3α and GSK3β at inhibitory residues. GSK3 signaling has a complex role in cancer biology, contributing to cell proliferation, metabolic regulation, and survival [47]. Inactivation of GSK3 may disrupt oncogenic signaling pathways and further contribute to growth suppression, as reported in lung carcinoma where it is considered a good prognostic factor [48]. Together, these findings support a model in which OLE induces a multifaceted stress response that culminates in inhibition of cell proliferation, checkpoint and caspase activation, and consequently apoptotic cell death in sensitive SCLC cells. In this context, the contribution of additional mechanisms, such as an increase in intracellular reactive oxygen species (ROS), cannot be excluded, since we have already reported this feature in neuroblastoma [28]. A strikingly different molecular scenario emerged in the SKMES NSCLC cell line. Despite evidence of reduced proliferation, OLE failed to induce significant activation of caspase-3, -7, or -9, and no relevant apoptotic response was detected. Nevertheless, antibody array analyses revealed extensive modulation of proteins involved in oxidative stress regulation and cell survival, suggesting that SKMES cells activate compensatory mechanisms capable of counteracting OLE-induced stress. Among the most relevant changes, OLE induced the expression of catalase and clusterin. Catalase represents a major anti-oxidant enzyme responsible for hydrogen peroxide detoxification and may therefore limit the accumulation of reactive oxygen species generated following OLE exposure, as expected by the well-known anti-oxidant activity of this compound [49]. Clusterin has been widely associated with treatment resistance, cellular stress adaptation, and tumor survival [50,51]. The up-regulation of both proteins suggests that SKMES cells efficiently activate protective anti-oxidant pathways, thereby preventing the propagation of pro-apoptotic signals. The resistant phenotype of SKMES cells was further supported by the increased expression of cIAP-2 and Bcl-xL, two potent anti-apoptotic molecules [52,53]. cIAP-2 negatively regulates caspase activation, whereas Bcl-xL preserves mitochondrial integrity and antagonizes mitochondrial apoptosis. The induction of these proteins provides a plausible explanation for the absence of caspase activation observed in SKMES cells and suggests that OLE-induced stress is effectively neutralized before reaching the threshold required for apoptotic execution. In addition, OLE promoted activation of STAT3, ERK1/2, and eNOS signaling pathways, that are well-recognized mediators of tumor cell survival, proliferation, and resistance to therapy [54,55,56]. Constitutive activation of STAT3 and ERK signaling has been associated with poor prognosis and therapeutic resistance in lung cancer [55,57]. Their induction following OLE treatment suggests the activation of adaptive feedback loops that may protect SKMES cells from cell death. Interestingly, HIF-1α was also up-regulated, indicating the possible engagement of metabolic adaptation programs that support survival under stressful conditions [58]. Collectively, these findings suggest that, unlike GLC-1 cells, SKMES cells respond to OLE by activating a predominantly cytoprotective transcriptional and signaling network.

The comparison between GLC-1 and SKMES cells therefore provides important mechanistic insights into the determinants of OLE responsiveness. Although both cell lines perceive OLE as a source of cellular stress, the downstream consequences are profoundly different. We may hypothesize that such different responses might be related to the different molecular characteristics of these two cell lines. GLC-1 cell line carries the chromosome 3p deletion along with loss of p53 and RB1, a classical genetic double-hit found in SCLC, which may lead to tumor progression [38]. In such cells, stress signaling is coupled to inhibition of cell proliferation, activation of apoptosis, ultimately resulting in cell death, and such effects may be at least in part related to these genetic and molecular alterations. SKMES cell line also carries a high mutation rate of p53, along with the expression of Mesenchymal–Epithelial Transition (MET) and Recepteur d’Origine Nantais (RON) tyrosine kinases and losses or rearrangements in different chromosomes. In addition SKMES, upon exposure to drugs, upregulate ATP-binding cassette transporters like P-glycoprotein MDR1, which actively pumps these drugs out of the cell [59]. These cells also appear capable of counter-balancing OLE-induced stress through coordinated activation of anti-oxidant defenses, anti-apoptotic proteins, and pro-survival signaling pathways. These observations suggest that the balance between stress-inducing and adaptive responses, along with molecular and genetic features, may represent a key determinant of the response to OLE treatment.

Noteworthily, OLE, in this treatment-resistant cell line, was surprisingly able to impair tumor cell migration. This result supports the pleiotropic anti-cancer activity of OLE. Although the molecular mechanisms underlying this effect were not specifically investigated, modulation of stress-responsive pathways and inhibition of pro-tumorigenic signaling networks may contribute to reduced migratory potential. Furthermore, the down-regulation of β-catenin, observed in NSCLC cells upon OLE treatment, may be related to the reduced migration of these cells. Indeed, the activation of β-catenin signaling has been previously described in the migration of epithelial and tumor cells [60,61]. Future studies will explore this issue, addressing whether the modulation of β-catenin is a cause or a consequence of the impaired migration and whether OLE affects additional anti-tumor mechanisms.

Other studies addressed potential anti-tumor effect of plant-derived extracts on lung cancer cells. In particular, essential oils extracted from *Croton tiglium*, *Cymbopogon citratus*, *Origanum majorana*, *Vitex agnus-castus*, *Eucalyptus tereticornis* and many others exerted different anti-cancer activities on NSCLC cell lines, including inhibition of cell proliferation, block of cell cycle, induction of apoptosis, modulation of cellular pathways and reduction in cell migration [62]. Similar effects on NSCLC were observed using extracts from *Zataria multiflora* and *Nigella sativa*, either on cell lines in vitro or in animal models [63], and using extracts from different medicinal plants [64]. Interestingly, all these compounds were mostly active on NSCLC models, whereas OLE demonstrated similar effects but predominantly on SCLC cell lines.

Another relevant finding of the present study, especially in clinical perspectives, is the synergistic interaction between OLE and cisplatin. Synergy analyses demonstrated that OLE significantly enhanced cisplatin-induced apoptosis in GLC-1 cells and quite surprisingly in SKMES cells, whereas no synergistic effect was observed in GLC-4 and A549 cell lines, thus suggesting that such synergy might be related to intrinsic molecular characteristics of cell lines, which may be similar in GLC-1 and SKMES cells, irrespective of their different tumor origin (SCLC or NSCLC). Since activation of DNA damage checkpoints and apoptosis emerged as prominent mechanisms of OLE activity, it is conceivable that OLE increases cisplatin-induced cytotoxicity, as reported in other cancers using chemotherapeutic agents [27,28]. The results obtained in our study appear to be particularly interesting in view of a future hopeful clinical application. From this standpoint, pharmacokinetic and bioavailability profiles of active compounds present in OLE must be taken into consideration. Notably, this issue has been addressed by previous studies, where oleuropein and hydroxytyrosol have been proved to be stable after digestion, with a plasmatic peak 20–75 min after OLE ingestion. Noteworthily, these two compounds are still present after 2–3 h [65,66]. Based on the data collected in the above studies, it is apparent that the plasma concentration of the OLE’s active compounds cannot equal the ones effective in in vitro testing. For this reason, it has to be pointed out that in vivo experimentation, aimed at identifying the effective and safe dose, is mandatory. On the other hand, it is to be underlined that the results obtained in drug combination studies show synergistic effects even at the lowest doses of OLE used. In view of a clinical application, it seems reasonable to exploit drug delivery systems (DDS, e.g., nanoparticles, liposomes, EVs) to try to overcome the limitation that might arise from low bioavailability [67]. Moreover, the functionalization of DDS to specifically target tumor cells could also be pursued.

## 5. Conclusions

In conclusion, this study identifies OLE as a pleiotropic modulator, with differential responses between sensitive and resistant cell lines contributing to its effects. In SCLC models, OLE affected cellular proliferation, apoptosis, and stress–response pathways. The enhancement of cisplatin-induced apoptosis observed in vitro suggests that OLE may warrant further investigation as a potential adjunct to chemotherapy. The latter effect may be related to intrinsic molecular characteristics of LC cells, irrespective of their histological category. Although OLE has an established history of use as a nutraceutical and is recognized as GRAS (Generally Recognized as Safe) by the FDA, these findings should be interpreted within the limitations of the present in vitro study. Further studies in appropriate in vivo preclinical models are needed to determine whether the observed effects can be reproduced in vivo, as well as to characterize OLE pharmacokinetics and its potential interactions with chemotherapeutic agents. Such evidence will be necessary to assess whether OLE could have a role as a dietary adjunct to chemotherapy before any consideration of clinical application.

## Figures and Tables

**Table 1:** Inhibition of cell proliferation by OLE (expressed as % of inhibition).

Cell Line	OLE (μM)				IC50
	50	100	200	400	
A549	3.26	7.11	20.40	90.02	233.57
CALU-6	26.11	31.92	59.42	100.00	125.26
GLC-1	44.04	65.99	71.70	61.55	39.01
GLC-4	3.62	54.80	98.31	100.00	105.24
GLC-82	11.79	33.15	27.82	100.00	169.65
SKMES	4.67	9.98	14.42	56.07	495.83

**Table 2:** Induction of early (left) and late (right) apoptosis by OLE after 48 h (as % of Annexin V^+^ cells or Annexin V^+^/7AAD^+^ cells, respectively).

Cell Line	OLE (μM)				OLE (μM)			
	50	100	200	400	50	100	200	400
A549	3.146	4.792	6.522	14.84	0.654	3.362	10.47	12.68
CALU-6	0.9767	2.132	20.45	27.71	1.583	1.687	5.148	24.27
GLC-1	1.344	4.252	12.29	2.042	2.012	8.558	32	64.64
GLC-4	3.018	6.926	9.68	14.43	1.866	4.166	7.204	13.71
GLC-82	1.027	1.343	16.7	20.36	1.507	3.442	10.56	16.99
SKMES	2.162	4.585	11.39	5.978	0.7733	2.06	5.695	8.422

## Data Availability

Data supporting reported results are available upon request from the corresponding author.

## Abbreviations

The following abbreviations are used in this manuscript:

SCLC	Small Cell Lung Cancer
NSCLC	Non-Small Cell Lung Cancer
CFSE	Carboxyfluorescein diacetate N-succinimidyl ester
CTR	Control
OLE	Olive leaf extract
h	hours
min	minutes
BrdU	bromodeoxyuridine
7AAD	7-amino-actinomycin D
HSP	Heat-shock protein
cIAP	cellular inhibitor of apoptosis
HIF-1α	hypoxia-inducible factor 1α
STAT3	Signal Transducer and Activator of Transcription 3
ERK1/2	Extracellular signal-Regulated Kinases 1/2
eNOS	endothelial Nitrox Oxigen synthase
NSA	neuron-specific enolase
CK-BB	BB isoenzyme of creatine kinase
MET	Mesenchymal–Epithelial Transition
RON	Recepteur d’Origine Nantais
DDS	drug delivery systems
GRAS	general recognized as safe
ROS	reactive oxygen species
